# Cancer Cell Adhesion and Metastasis: Selectins, Integrins, and the Inhibitory Potential of Heparins

**DOI:** 10.1155/2012/676731

**Published:** 2012-02-12

**Authors:** Gerd Bendas, Lubor Borsig

**Affiliations:** ^1^Department of Pharmaceutical Chemistry, University of Bonn, 53121 Bonn, Germany; ^2^Institute of Physiology, University of Zürich and Zürich Center for Integrative Human Physiology, 8057 Zürich, Switzerland

## Abstract

Cell adhesion molecules play a significant role in cancer progression and metastasis. Cell-cell interactions of cancer cells with endothelium determine the metastatic spread. In addition, direct tumor cell interactions with platelets, leukocytes, and soluble components significantly contribute to cancer cell adhesion, extravasation, and the establishment of metastatic lesions. Clinical evidence indicates that heparin, commonly used for treatment of thromboembolic events in cancer patients, is beneficial for their survival. Preclinical studies confirm that heparin possesses antimetastatic activities that lead to attenuation of metastasis in various animal models. Heparin contains several biological activities that may affect several steps in metastatic cascade. Here we focus on the role of cellular adhesion receptors in the metastatic cascade and discuss evidence for heparin as an inhibitor of cell adhesion. While P- and L-selectin facilitation of cellular contacts during hematogenous metastasis is being accepted as a potential target of heparin, here we propose that heparin may also interfere with integrin activity and thereby affect cancer progression. This review summarizes recent findings about potential mechanisms of tumor cell interactions in the vasculature and antimetastatic activities of heparin.

## 1. Introduction

**Figure 1:**
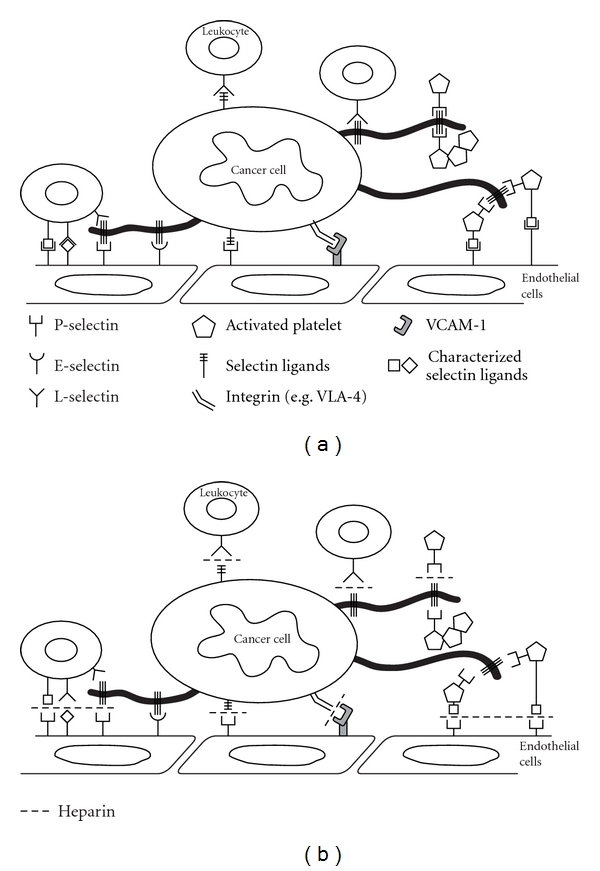
Selectins and integrins contribute to metastatic spread. (a) Schematic presentation of selectin- and integrin-mediated cancer cell interactions with several blood constituents (e.g., platelets, leukocytes, and endothelial cells) during hematogenous metastasis. (b) Heparin application in mouse models blocks both P- and L-selectin-mediated; and VLA4-mediated interactions of cancer cells within blood circulation thereby affecting metastasis.

Metastasis is facilitated by cell-cell interactions between tumor cells and the endothelium in distant tissues. Tumor cells in circulation interact also with platelets and leukocytes that further contribute to tumor cell adhesion, extravasation, and the establishment of metastatic lesions. Hematogenous cancer metastasis is a multistep cascade encompassing process, starting with local invasion of tumor cells at primary tumors, survival in systemic circulation, extravasation in secondary sites, and ending with establishment of growing metastatic lesions. The metastatic capacity of tumor cells correlates with their ability to exit from the blood circulation, to colonize distant organs, and to grow in distant organs [1]. The organ-specific character of metastasis has been already observed by S. Paget more than a century ago, and the “seed and soil” hypothesis postulates specific interactions of tumor cells with the “friendly” environment of distant organs that enables the establishment of metastasis and subsequent growth [2]. While cell-cell interactions facilitating tumor cell adhesion in the vasculature of specific organs are essential steps in the metastatic cascade, inhibition of these interactions represent a therapeutically useful target for attenuation of metastasis (Figure 1). Two major cell adhesion molecule families, selectins and integrins, have been identified to participate in metastasis [3–6]. In addition to direct targeting of selectin and integrins, recent evidence suggests that heparin possesses selectin (P- and L-selectin) and also integrin inhibitory activity [7–9]. Heparin and low-molecular-weight (LMW) heparin are commonly used anticoagulants for treatment of cancer-associated thromboembolism in cancer patients [10, 11]. The exact mechanism of cell-cell interactions involved in cancer progression remains to be fully elucidated; however, the potential of heparins to interfere with this process warrants further investigations.

## 2. Cell Adhesion as a Determinant of Metastasis

Within blood vessels, circulating tumor cells ultimately interact with the endothelium that might lead to tumor cell arrest and extravasation (Figure 1). Studies of tumor cell-endothelial contact formations have been based on parallels to the leukocyte-endothelial cell interactions during inflammation. Although the mechanism of tumor cell adhesion certainly differs from leukocyte recruitment to inflammatory sites, the cell adhesion molecules involved in the contact formation with endothelium are potentially the same [3, 5, 6]. Indeed, there is accumulating evidence for the role of selectins and integrins in cancer progression of various cancer types, including colon and lung carcinomas and melanomas [5, 6]. While selectin-mediated tumor cells arrest and adhesion contribute to metastasis, integrin-mediated interaction from both tumor cells and the surrounding environment further contribute to cancer progression.

## 3. Selectins

Selectins are vascular cell adhesion molecules involved in adhesive interactions of leukocytes and platelets and endothelium within the blood circulation. There are three members of the selectin family: P-, E-, and L-selectin. P-selectin is present in the storage granules of platelets (*α*-granules) and endothelial cells (Weibel-Palade bodies), thus enabling rapid translocation on cell surfaces upon activation [12]. On the contrary, endothelial expression of E-selectin requires *de novo* transcription, leading to expression on activated endothelial cell surfaces several hours after stimulation [12]. L-selectin is constitutively expressed on cell surfaces of almost all leukocyte subpopulations. The physiological functions of selectins are well described in processes of inflammation, immune response, wound repair, and hemostasis [13]. The role of selectins in these processes has been elucidated in mouse deficient in individual selectins. Whereas L-selectin mediates fast rolling of leukocytes on endothelium, P- and E-selectins support rolling at lower velocities [13]. The initial steps in leukocyte tethering and rolling on endothelium are supported by rapid and reversible interactions of selectins with their carbohydrate ligands.

The majority of selectin ligands consist of distinct glycan structures carrying the terminal core tetrasaccharide structure sialyl Lewis^x/a^ (sLe^x^/sLe^a^) on a protein backbone [14, 15]. Selectins binds to various classes of molecules (mucins, sulfated glycolipids, glycosaminoglycans), and most of these molecules were shown to be functional selectin ligands *in vivo* [12]. Efficient selectin binding to carbohydrates usually requires a proper glycoprotein scaffold that presents several selectin ligands in clusters, thereby increasing the avidity of the interaction.

### 3.1. Selectins as Facilitators of Metastasis

Hematogenous metastasis is the common route for cancer spread of epithelial cancers—carcinomas. In the normal physiological state, epithelial cells line the lumen of hollow organs and are covered by mucins that are either cell surface attached or building soluble layers covering the epithelium. Mucins are high-molecular-weight molecules with a large proportion of O-linked glycans attached to a protein backbone. During malignant transformation cell surface glycans undergo dramatic changes [16]. The major alterations of glycans on tumor cells are associated with enhanced expression of sLe^x^ or its isomer sLe^a^, Tn and sialyl-Tn antigen structures [16, 17]. Enhanced expression of sLe^x^ and sLe^a^ structures is frequently associated with progression and poor prognosis in various cancers including colon, gastric, lung, renal, and breast cancers, melanomas, and others (reviewed in [18]). Several laboratories have shown that at least one selectin (P-, L-, or E-) is capable to bind to any human carcinoma tested (e.g., [19, 20]), which emphasize the potential of selectins to mediate contacts with tumor cells within vasculature. The fact that, during the hematogenous phase of metastasis, selectin ligand-carrying tumor cells may encounter selectins, present on blood constituents (leukocytes, platelet, and endothelium) in the circulation, supported the notion of selectin involvement in metastatic progression [3, 5]. This hypothesis has been evaluated by several laboratories, and recent findings using mouse models deficient in one or more selectins confirmed the involvement of P-, L-, and also E-selectin in metastasis [7, 21–24].

### 3.2. P-Selectin and Metastasis

The rapid expression of P-selectin on cell surfaces of endothelial cells and platelets upon activation makes P-selectin a likely candidate involved in the metastatic process. There is accumulating evidence that formation of platelet-tumor cell thrombi helps evading host responses, thereby contributing to metastasis [7, 25–27]. In the absence of platelet-tumor cell interactions, tumor cells are cleared by NK cells [26, 28]. Minimal platelet-tumor cell microthrombi has been detected in the absence of P-selectin, leading to reduced tumor cell adhesion in the lungs of mice and subsequently attenuation of metastasis [7, 22, 29]. Furthermore, removal of cell surface mucins from tumor cells prior to intravenous injection resulted in reduction of metastasis [7]. Bone marrow reconstitution experiments in P-selectin-deficient mice have shown that endothelial P-selectin, in addition to platelet P-selectin, contributes to metastasis [29].

Patients with metastatic carcinoma cancer are at high risk for thromboembolic events, a finding initially described by Trousseau [30]. The association between mucinous carcinoma and Trousseau's syndrome led to the hypothesis that mucin might directly induce thrombi formation [31]. Intravenous injection of purified carcinoma mucin led to thrombi formation that was dependent both on the presence of P- and L-selectin. Recently, the molecular mechanism of mucin-induced initiation of tumor cell-platelet complexes has been described [32]. Microthrombi formation induced by carcinoma mucins was found to be dependent on L-selectin and PSGL-1 expression on neutrophils that induced cathepsin G release thereby triggering platelet activation and P-selectin expression.

### 3.3. L-Selectin and Metastasis

Participation of leukocytes in platelet-tumor cell emboli is well described, yet the role of leukocytes in the process of metastatic initiation remains under investigation. The establishment of a metastatic niche is based on the initial recruitment of bone marrow-derived cells to distant sites in organs where metastatic cells tend to seed [33, 34]. In general, contribution of leukocytes to metastasis largely depends on spatial and temporal situation that is defined by the microenvironment and tumor cells [34–36]. Whether L-selectin mediates leukocyte recruitment to metastatic sites has been tested in L-selectin-deficient mice [21, 37]. The absence of L-selectin led to significant attenuation of metastasis, indicating that L-selectin actively contributes to leukocyte recruitment and formation of a metastatic niche [5, 38]. Reduced numbers of CD11b-positive leukocytes has been detected at early time points after tumor cell injection that correlated with reduced tumor cell survival in the lungs [37]. Recent evidence indicates that selectin-mediated interactions through cooption of inflammatory pathways contribute to formation of a permissive microenvironment for metastasis [38]. Tumor cell-mediated activation of the adjacent endothelium upon vascular arrest resulted in NF-*κ*B activation and expression of E-selectin, thereby contributing to metastasis.

### 3.4. E-Selectin and Metastasis

E-selectin has been investigated as a mediator of metastasis at sites where arrest of tumor cells in the microvasculature has been observed [39, 40]. E-selectin expressed on activated endothelial cells has been detected during metastatic colonization of the liver [39, 41]. Inhibition of E-selectin or downregulation of E-selectin expression resulted in attenuation of experimental liver metastasis. In contrast, transgenic overexpression of E-selectin in the liver redirected metastasis to this organ, thereby confirming the role of E-selectin in this process. Interestingly, experimental metastasis of human colon carcinoma cells lines was not affected by the absence of E-selectin [24]. However, spontaneous breast metastasis to the lung was reduced in E-selectin-deficient mice, indicating E-selectin involvement during lung metastasis [42]. Organ-specific differences may contribute to the colonization process and different requirements for selectin-mediated interactions may be dependent on the primary tumor and the metastatic organ.

While selectins were identified as potential facilitators of metastasis, they have not been explored as pharmacological targets for treating cancer progression.

## 4. Biology of Integrins

Integrins are large and complex transmembrane glycoproteins. The structure of integrins consists of two distinct chains, *α*- and *β*-subunit, which form a non-covalent heterodimer [43, 44]. In mammals, 18 *α*- and 8 *β*-integrins have been characterized that combine to form 24 unique canonical *α*/*β* receptors identified so far. Integrins mediate cell adhesion and directly bind components of the extracellular matrix (ECM), such as fibronectin, vitronectin, laminin, or collagen, thereby providing anchorage for cell motility and invasion. Since specific integrins can bind to different ligands and identical ligand can be shared by different integrins, this redundancy underscores the general importance of integrins in cell communication.

Integrins are mediators of a bidirectional signaling where intracellular signals induce alterations in the conformation, thus ligand-binding properties (inside—out signaling). Since integrins are linked to cytoskeletal structures (e.g., **α**-actinin, talin, and vinculin) ligation of extracellular ligands can influence intracellular processes (outside—in signaling) through activation of kinases, GTPases of the Ras/Rho signaling pathways [44, 45]. The convergence between the cytoskeleton and ECM components is mediated via a cluster formation of integrins and their downstream signaling molecules, focal adhesion kinase (FAK) or Src family kinases, which affect the cellular shape and migratory properties of cells [46]. In addition to the well-established role of integrins during migration and invasion, integrins also regulate cell proliferation, survival and angiogenesis, all processes actively investigated during cancer progression.

## 5. Integrins during Cancer Metastasis

The ubiquitous presence of integrins on tumor cells, blood components, vasculature, and stromal cells suggest that integrins might essentially contribute to different steps in the metastatic cascade. As many human tumors originate from epithelial cells, integrins expressed on epithelial cells are generally present also in tumor cells. Studies correlating integrin expression levels with the pathological outcomes, such as metastasis or patient survival, have identified several integrins that might be involved in cancer progression [6]. Tumor cell expression of *α*v*β*3, *α*v*β*5, *α*5*β*1, *α*6*β*4 correlated with metastatic progression in melanoma, breast carcinoma, prostate and pancreatic and lung cancer [6].

During hematogenous phase of metastasis tumor cell platelet interaction are mediated either by P-selectin (see above) or through platelet integrin *α*IIb*β*3. Inhibition of *α*IIb*β*3 integrin or P-selectin by function-blocking antibodies significantly reduced platelet-tumor cell interaction and tumor cell adhesion on activated endothelium [47–49]. Accordingly, attenuation of metastasis has been observed.

Tumor cell expression of *α*v*β*3 integrin together with its capacity to bind several ECM components, including fibronectin, vitronectin, and osteopontin, has been regarded as critical factor for affecting the site of metastasis. In this respect, fibrinogen was identified as a bridging factor between *α*IIb*β*3 integrins on platelet and *α*v*β*3 on tumor cells [50, 51]. This interaction facilitates tumor cell arrest in the vasculature and metastasis to various tissues, including bone marrow and lungs.

The contribution of integrins to angiogenesis and thereby tumor progression and metastasis has recently been reviewed [52]. Tumor-associated vessels express *α*v*β*3 and *α*v*β*5 integrins that were not detected in quiescent vasculature [53]. The binding of these vascular integrins to ECM components in the tumor microenvironment contributes to invasion and migration of endothelial cells. Therefore, targeting of *α*v*β*3 and *α*v*β*5 integrins with antibodies or peptide- (RGD-) derived structures has been investigated as a promising antiangiogenic approach.

The establishment of metastatic niche is dependent on the recruitment of bone marrow-derived cells [33, 34]. Homing of circulating progenitor cells to tumors was shown to require *α*4*β*1 integrins [54]. Expression of integrin *α*4*β*1 (VLA-4) on bone marrow-derived cells mediates binding to VCAM and cellular fibronectin, which are present at sites of endothelial remodeling.

Initial studies on the role of integrins during metastasis, specifically investigating primary endothelial contacts, were focused on melanoma metastasis [55–57]. Metastatic dissemination of murine melanoma B16F10 cells has been blocked by peptide displaying the RGD integrin-binding sequence of fibronectin [55]. The targeted integrin involved in the vascular arrest of melanoma cells has later been identified to be VLA-4 [56, 57]. Experimental pulmonary metastasis of melanoma cell lines, B16 and A 375 M, was confirmed to be mediated by VLA-4. The specific VLA-4-mediated interaction with VCAM-1 on endothelium is required for melanoma cell adhesion and endothelial transmigration [58]. Recently, VLA-4 mediated melanoma adhesion to VCAM-1 on activated endothelium was shown to support extravasation under the shear flow also in the absence of selectin ligands [59], indicating the potential of VLA-4 to serve as an adhesion molecule during metastatic spread of cancer.

Besides the role of VLA-4 in melanoma metastasis, *β*1 integrins strongly contribute to metastasis of other tumor types, for example, lymphomas [60]. Silencing of *β*1 integrins in the highly metastatic Esb murine T-lymphoma cell line, and thus the loss of VLA-4 and VLA-6 binding, strongly reduced the metastatic dissemination to the lungs and spleen. Furthermore, a change in metastatic pattern with prevalence for skeletal muscle invasion has been observed [60]. To investigate the role of *β*1 integrins during tumor growth and metastasis, a fibroblastoid cell line with disrupted *β*1 integrin gene has been generated [61]. Overexpression of *β*1 integrin significantly correlated with metastatic spread of these cells to the lung and liver, when compared with parental cells with disrupted *β*1 integrin gene [61]. Taken together, integrins are becoming attractive therapeutic targets for therapeutic strategies focused not only on tumor development but also on metastasis.

Since heparins effectively block both P-, L-selectins and VLA-4 integrin-mediated tumor cell adhesion, heparin and heparin derivatives have been tested in a number of animal models for their potential to attenuate metastasis (reviewed in [9]).

## 6. Heparin and Cancer: Clinical Evidence

Heparin is commonly used for the prevention or treatment of venous thromboembolism in cancer patients. In addition to its antithrombotic activity, cancer patients treated with heparins showed an improved survival in a number of retrospective and prospective studies (reviewed in [62, 63]). A recent review on antithrombotic therapy using heparins concluded that, despite the heterogeneity of completed clinical studies, heparin treatment of cancer patients with better prognosis is beneficial for patients primarily due to prolonged survival [10]. Based on this conclusion, together with observations from animal models, heparin appears to directly affect cancer progression associated with metastatic spread.

## 7. Heparin Attenuates Metastasis through Inhibition of Selectins

Heparin is a complex mixture of natural glycosaminoglycans based on repeating disaccharide units containing glucosamine and glucuronic/iduronic acid residue with a high degree of sulfation [64]. Heparin and LMWH were tested in many different animal models for their potential to inhibit cancer progression primarily using experimental metastasis models (reviewed in [65, 66]). Despite large variation in heparin preparations, doses applied, time of application, and different tumor models, attenuation of metastasis has been observed almost in all independent studies when heparin was applied around the time of tumor cell injection. Together with the limited effect on tumor growth [67, 68], these findings indicate that heparin affects processes when tumor cells are still in the blood circulation. Because of the very nature of heparin, several other biological activities in addition to the anticoagulant activity have been detected such as binding to cell adhesion molecules (P- and L-selectin, VLA-4 integrin), enzymes (heparanase), growth factors, and cytokines [69–73]. Chemically modified heparins were prepared and tested for various biological activities [71]. Modified heparins containing mostly P-selectin inhibitory activity were shown to efficiently attenuate metastasis almost to the same levels as observed in P-selectin-deficient mice [71]. Since heparin injection in P-selectin-deficient mice resulted in no further attenuation of metastasis, these findings indicated that heparin affects metastasis by inhibition of P-selectin [7, 29, 71]. Interestingly, a single dose of heparin prior to tumor cell injection further attenuated metastasis in L-selectin-deficient mice, indicating that L-selectin involvement in this process is subsequent to P-selectin [21]. Further evidence for sequential involvement of P- and L-selectin in metastasis was confirmed by observation that L-selectin contributes to metastasis first several hours after tumor cell injection [37]. Heparin injection 6–12 hours after the tumor cell challenge further reduced metastasis in P-selectin-deficient mice confirming the potential of heparin to inhibit also L-selectin-mediated interactions. Taken together, these findings indicate that inhibition of P- and L-selectin attenuates metastasis and heparins appear to be efficient inhibitors of selectin mediated interactions *in vivo*.

## 8. Heparin Binding and Inhibition of Integrin Functions

Heparin as a potential inhibitor of integrin-mediated cell-cell interaction has been evaluated only in few studies. The *α*M*β*2 (Mac-1) integrin on hematopoietic progenitor cells was shown to mediate adhesion to stromal compartment through binding to heparin and heparan sulfate [74, 75]. Another study described the leukocyte integrin *α*X*β*2 to bind sulfated heparin in a low micromolar range [76]. These studies suggest that heparin can interfere with leukocyte binding and recruitment to the endothelium.

Zhang et al. reported that platelet integrin *α*IIb*β*3 can efficiently be blocked by heparin and non-anticoagulant heparin derivatives [77]. Platelet interaction with melanoma cells A375 or B16F10 was strongly reduced *in vitro* and metastasis *in vivo*. These findings indicate that heparin inhibition of *α*IIb*β*3 integrins is additional to P-selectin inhibition of platelet binding (as mentioned above) with relevance for those tumors cells with low expression levels of P-selectin ligands.

Heparin inhibition of integrin-mediated melanoma adhesion to endothelium has been reported only recently [73]. Expression of integrin *α*4*β*1 (VLA-4) by B16F10 melanoma cells mediated their adhesion to endothelial cells through binding to VCAM-1. Heparin was shown to inhibit the VLA-4 mediated melanoma binding to VCAM-1 substrates under dynamic conditions. A follow-up study using human melanoma MV3 cells confirmed heparin binding to VLA-4 with binding affinities in the low micromolar range [78]. Structural analysis of heparin indicated a size dependency of integrin binding, since short heparin fragment or the pentasaccharide (Fondaparinux) was not able to bind VLA-4 [8]. Further analysis revealed that also the sulfation density is critical for VLA-4 recognition [79].

Altogether, these studies indicate that heparin can affect important steps in the metastatic cascade by inhibition of integrins. The relevance of this contribution depends on tumor cell types carrying integrin and/or selectin ligands. Melanoma cells with their high expression levels of VLA-4 appear especially relevant for this consideration. However, the efficiency to inhibit metastasis *in vivo* remains to be analyzed.

## 9. Tumor Cell Seeding and the Establishment of Metastatic Niche

Beside direct heparin inhibition of adhesion receptor functions, heparin can affect the activities of cellular proteoglycans related to cell adhesion. This possibility will shortly be introduced below with respect to potential antimetastatic approaches or novel targets.

Chemokines are chemotactic cytokines that induce direct migration of leukocytes to sites of inflammation or cancer progression [80]. Beside the well-described role of chemokines during inflammation, seeding of tumor cells to distant tissues was shown to be facilitated by chemokines.

There is compelling evidences that chemokine receptors, for example, CXCR4, mediate breast cancer metastasis [81]. Breast cancer cells expressing CXCR4 in circulation effectively enter the bone marrow niche due to enhanced expression of CXCL12 in this environment [82]. Targeted metastasis to the bone marrow or other sites with high expression of CXCL12 has been described in a number of cancers including breast, colon, and prostate [80]. Chemokines bind to glycosaminoglycans chains of proteoglycans presented on surfaces of epithelial and vascular endothelial cells or on extracellular matrix molecules. Cell migration is dependent on chemokine gradient presented by chemokines at specific sites [83]. Recently, syndecan-1 and syndecan-4 proteoglycans were shown to be required for chemokine- (CCL5-) induced hematoma migration and invasion [84]. Similarly, CCL2-induced human hepatoma cell migration and invasion has been blocked by anti-syndecan-1 and -4 antibodies, but also when hepatoma cells were pretreated with heparitinases that remove glycosaminoglycans from cell surfaces [85]. Interestingly, a recent study reported on the ability of LMW heparin to bind SDF-1 (CXCL-12) in a sub-micromolar range [86]. Since proper chemokine presentation by endogenous proteoglycans is a prerequisite for successful metastasis, heparin treatment might “remove” chemokine as a decoy and thereby reduce tumor cell adhesion and recruitment to the metastatic sites. Chemokine binding to its chemokine receptor could directly activate integrin binding function by inducing the conformational change of the integrins [44]. Whether heparin binding to chemokines indeed attenuates metastasis remains to be explored.

Fibronectin, an ECM protein, possesses specific and partly overlapping binding sites for the integrin VLA-4, VLA-5, and heparin [87]. The anchorage of cells to heparin-binding domains of fibronectin or other ECM components is mainly linked to syndecans. Several studies point to the critical role of the syndecan extracellular domains in tumor cell adhesion and invasion behavior where syndecan-4 acts as a “coreceptor” with integrin VLA-5, but not VLA-4 [43, 88]. Syndecan-4 binds to the extracellular matrix and is also connected to the actin cytoskeleton via interaction with structural and signaling proteins such as FAK, syndesmos, and paxillin. This represents both a mechanical and signaling link to cell surface integrin VLA-5 required for focal adhesion and stress fiber formation in cells adherent to fibronectin [89, 90]. Thus, the potential heparin antimetastatic activity can be based on interference either directly with the integrin-fibronectin binding or indirectly via competing for the proteoglycan binding of syndecan-4 to fibronectin. However, a direct interference of heparin with this pathway in cell migration remains to be defined.

Integrins were also shown to bind cystein-rich protein 61—Cyr61 [91]. Cyr61 was first identified as a growth factor-inducible immediate early gene and belongs to the CCN family of matricellular proteins (CCN1). Elevated levels of Cyr61 have been correlated with increased breast adenocarcinoma, endometrial tumors, pancreatic cancer, or glioma malignancy [92–95]. Cyr61 has been reported to mediate numerous cellular processes, such as cell adhesion, cell survival, proliferation, enhancement of growth factor-induced DNA synthesis, and angiogenesis [96]. These effects result, at least in part, through a direct binding of Cyr61 to the extracellular regions of the integrin *α*v*β*3 thus activating these adhesion and signaling receptors [97]. However, Cyr61 has also a binding ability to *α*2*β*1, *α*3*β*1, *α*5*β*1, *α*6*β*1, and *α*M*β*2 [91]. Cyr61 overexpression in gastric cancer cell line AGS was shown to increase peritoneal dissemination through increased *α*2*β*1 integrin activity [92]. In contrast, Cyr61 silencing in PC-3 and DU-145 prostate cancer cells strongly inhibited proliferation [98]. Though, Cyr61 also significantly enhanced TRAIL-induced apoptosis through interaction with integrins *α*v*β*3 and *α*6*β*4 [98]. Therefore, inhibition of Cyr61 activity appears as a promising therapeutic approach to inhibit tumor cell growth, migration, and adhesion. The Cyr61 molecule has two discrete heparin binding sites which contribute to binding to cell surface on heparan sulfate proteoglycans on Syndecan-4 [91]. Consequently, heparin could indirectly influence integrin functions by depleting released Cyr61. Although, previous heparin treatment in a number of animal models might have affected also Cyr61 activity, further analysis is required to elucidate the relevance of this signaling pathway for antiadhesive approaches of heparin applications.

## 10. Conclusions

Accumulating evidence from several preclinical models confirms that tumor cell interactions through selectins and integrins actively contribute to the metastatic spread of tumor cells. However, the current cancer therapies are focused only on targeting of tumor cells while no specific therapy for inhibition of metastatic spread is available. Clinical findings suggest that heparin and LMW heparin possess anticancer activities leading to survival benefits for cancer patients in the early stage of the disease. Although the identification of underlying molecular mechanisms is still ongoing, several preclinical studies confirmed the dominant contribution of selectins to metastasis and their role as primary targets of heparin.

In addition, integrins were shown to contribute to cancer progression. Since heparin binds to particular integrins implicated in metastasis, targeting integrins opens additional ways for interference with metastatic progression in addition to inhibition of angiogenesis, cell migration, or establishment of the premetastatic niche.

The elucidation of the orchestrated functions of selectin and integrins and possibly other adhesion molecules during metastatic cascade requires further studies. Clearly further clarification of heparin interactions with selectins and integrins is required, yet the abundant clinical experience with heparin and LMW heparin proposes its evaluation as a potential antimetastatic treatment.
